# Phase Change Materials for Battery Thermal Management: From Material Synthesis to Hybrid Systems

**DOI:** 10.3390/nano16161030

**Published:** 2026-08-19

**Authors:** Sibo Yang, Lang Qin, Fangzheng Zhou, Xing Li, Hongsheng Dong

**Affiliations:** 1 School of Marine Science and Technology, Northwestern Polytechnical University, Xi’an 710072, China; 2 National Innovation Platform (Center) for Industry-Education Integration of Energy Storage Technology, Xi’an Jiaotong University, Xi’an 710049, China

**Keywords:** phase change materials, battery thermal management, thermal conductivity enhancement, flame retardancy, hybrid cooling systems, lithium-ion batteries

## Abstract

Effective thermal management is a cornerstone of safe, long-life lithium-ion battery operation, especially under high-rate charge–discharge and dynamic driving conditions. Conventional active cooling technologies face inherent trade-offs between heat dissipation efficiency, system complexity, and temperature uniformity, while phase change materials (PCMs) provide a promising passive alternative by absorbing latent heat during phase transition to buffer temperature spikes, improve temperature uniformity, and delay thermal runaway propagation. This paper presents a comprehensive review of recent advances in PCM-based lithium-ion battery thermal management, systematically covering the full scope from fundamental battery heat generation mechanisms to material synthesis optimization and hybrid system integration. At the material level, we analyze state-of-the-art strategies to address the intrinsic drawbacks of organic PCMs—low thermal conductivity, mismatched phase transition temperatures, and high flammability—including the construction of carbon/metal conductive skeletons, compositional tuning of phase change behavior, and flame-retardant modifications. These approaches have yielded composite PCMs with significantly improved heat transport capability and fire safety, while preserving high latent heat storage capacity. At the system level, we evaluate the thermal performance of pure passive PCM configurations, which excel at peak temperature suppression and inter-cell temperature uniformity, as well as hybrid designs that combine PCMs with air or liquid cooling to resolve heat accumulation issues and maintain stable performance under prolonged, demanding operating cycles. Despite these advances, key challenges remain: balancing high thermal conductivity with high latent heat capacity, developing climate-adaptable phase transition temperatures, and integrating multiple functionalities without compromising core thermal storage properties. Looking forward, future research directions include multifunctional integrated composites, smart adaptive PCMs, cost-effective scalable manufacturing, and precision structural engineering. This review also summarizes quantified performance trade-offs and provides actionable design guidelines for both material development and system-level integration.

## 1. Introduction

Lithium-ion batteries have established themselves as the dominant power source for electric vehicles and large-scale energy storage systems due to their high energy density, long cycle life, and relatively low self-discharge rates. For other electrochemical energy storage technologies such as vanadium redox flow batteries, functional nanomaterial-modified electrolytes have also been proven effective in enhancing electrochemical performance and operational stability, providing a reference for performance optimization of energy storage systems from the material perspective [1]. However, the performance, safety, and longevity of lithium-ion batteries are intrinsically linked to their operating temperature. Studies indicate that thermal management failures account for a significant proportion of lithium battery safety incidents, underscoring the critical need for effective thermal regulation systems [2]. The optimal operating temperature range for most commercial lithium-ion batteries is narrow, typically between 20 °C and 45 °C. Operating outside this window, whether in high-temperature or low-temperature environments, poses severe risks to battery integrity. Under high-temperature conditions (>45 °C), the electrochemical stability of the battery components deteriorates. Detrimental side reactions intensify, most notably the decomposition of the solid electrolyte interphase film on the anode surface [3]. The solid electrolyte interphase film is crucial for preventing further electrolyte decomposition; its breakdown leads to exothermic reactions between the electrolyte and the exposed anode material. Furthermore, elevated temperatures accelerate the oxidation of the electrolyte on the cathode surface. These reactions result in irreversible capacity loss and the generation of gas, which can cause swelling and increased internal pressure. If temperatures continue to rise and exceed 80 °C, a chain of exothermic reactions can be triggered, leading to thermal runaway—a catastrophic failure mode characterized by rapid temperature escalation, venting, fire, or explosion [4]. Accident statistics further corroborate that thermal management malfunction is a leading cause of these catastrophic events [5,6].

Conversely, low-temperature environments pose a different set of challenges. At sub-zero temperatures, the viscosity of the liquid electrolyte increases significantly, resulting in decreased ionic conductivity. This impedes the transport of lithium ions between the electrodes. During charging at low temperatures, the diffusion of lithium ions into the graphite anode is slower than the rate of lithium intercalation, leading to increased anode polarization [7,8]. When the anode potential drops below 0 V vs. Li/Li^+^, lithium ions are more likely to deposit on the anode surface as metallic lithium rather than intercalating into the graphite structure, a phenomenon known as lithium plating [9,10]. Lithium plating not only causes rapid capacity fading but also poses a severe safety risk as the plated lithium can form dendrites, which may penetrate the separator and cause internal short circuits.

In addition to absolute temperature control, temperature uniformity within a battery pack is paramount. Research indicates that temperature differences exceeding 5 °C between cells within a pack can accelerate the evolution of cell inconsistencies [11]. This non-uniformity leads to a phenomenon where some cells are overcharged or over-discharged relative to others, degrading the overall pack performance and lifespan. Therefore, an effective battery thermal management system must simultaneously meet dual requirements: maintaining the battery within the optimal temperature range and ensuring minimal temperature gradients across the battery pack.

Understanding the heat generation mechanism is fundamental to the design and optimization of thermal management systems. The operation of a lithium-ion battery is accompanied by heat generation, which can be attributed to both reversible and irreversible processes. The classical theory for quantifying battery heat generation was established by Bernardi et al., who proposed an energy balance model that expresses the battery’s heat generation power as the sum of reversible heat (entropic heat) and irreversible heat [12,13,14]. The reversible heat originates from the entropy change associated with the electrochemical reactions of lithium-ion intercalation and de-intercalation. This component can be either exothermic or endothermic depending on the state of charge and the direction of current flow (charging or discharging). In contrast, irreversible heat is always exothermic and constitutes the dominant portion of heat generation, particularly at high operating rates. Irreversible heat primarily consists of ohmic heat and polarization heat. Ohmic heat, also known as Joule heating, arises from the resistive losses during electron transport in the solid phases (electrodes, current collectors) and ion transport in the electrolyte. Polarization heat is generated due to the overpotentials associated with electrochemical reaction kinetics (activation polarization) and concentration gradients (concentration polarization).

Studies have quantified the contribution of these heat sources under various operating conditions. For instance, experimental measurements on commercial LiFePO_4_/graphite 18,650 cells confirm that at high discharge rates such as 3 C, irreversible heat accounts for over 70% of the total heat generation [15,16]. This dominance of irreversible heat at high currents necessitates rapid heat dissipation strategies to prevent thermal accumulation. To accurately predict the thermal behavior of batteries, researchers utilize electrochemical-thermal coupled models. These models resolve the interaction between microscopic electrochemical reactions and the macroscopic temperature field, providing insights into local hot spots and thermal gradients. Electro-thermal models, which are less computationally intensive, are more suitable for system-level thermal design. Relevant studies report that the error between model-predicted temperatures and experimental measurements can be within 2 °C, validating the accuracy of these modeling approaches [17]. Clarifying the heat generation mechanism provides the theoretical basis for selecting appropriate thermal management technologies and optimizing their design parameters.

**Figure 1:**
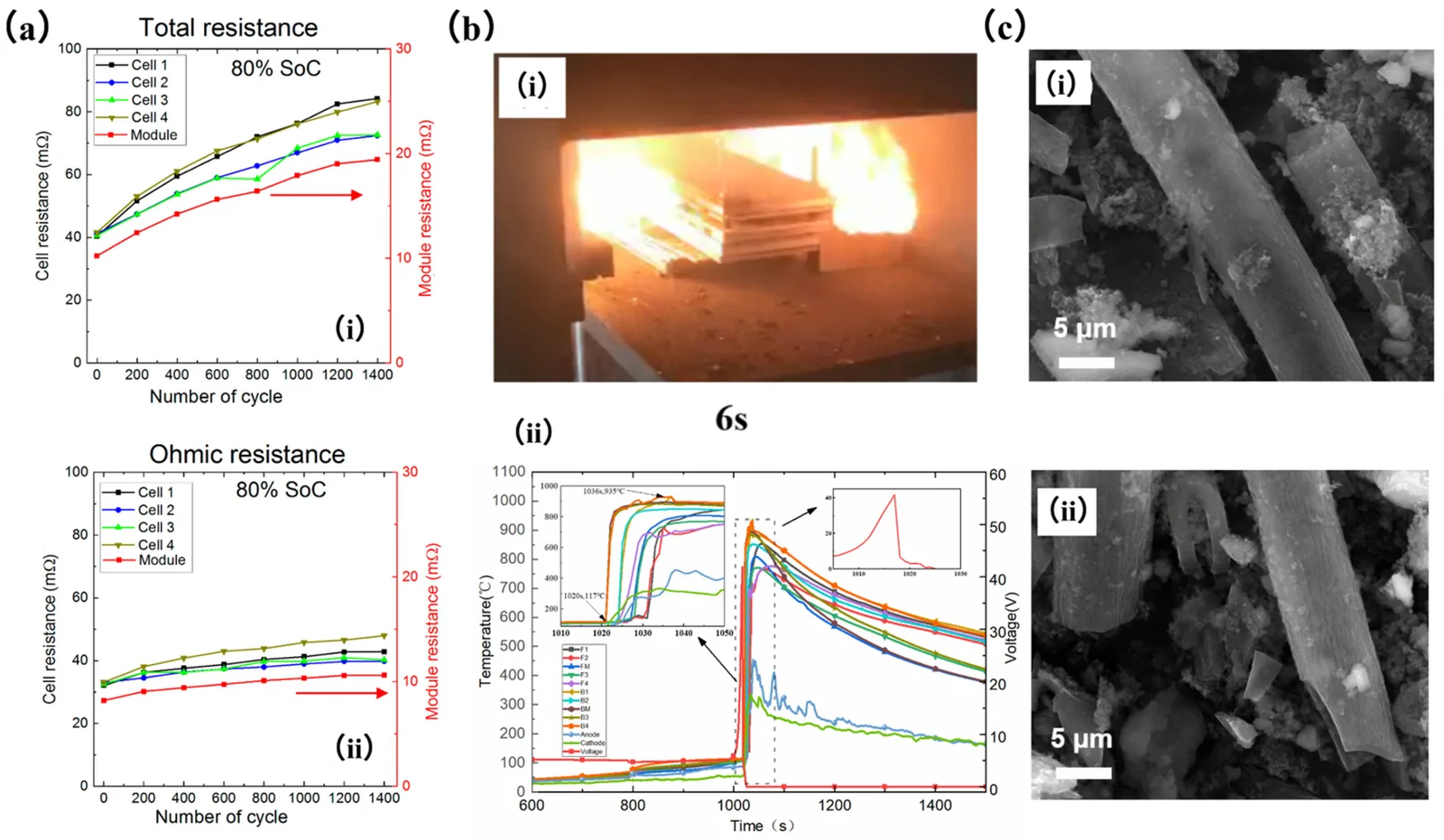
(**a**) Evolution of total resistance and ohmic resistance at 80% state of charge over 1400 cycles for parallel-connected cells, reflecting degradation inconsistency induced by temperature gradients. Reproduced from [18]. (**b**) Overcharge-triggered thermal runaway characteristics of a lithium-ion battery: representative flame image (upper) capturing the most violent ejection stage at the moment of thermal runaway, with synchronous temperature–voltage profiles (lower) showing the catastrophic temperature surge and voltage collapse. Flame image and temperature–voltage curves reproduced from [19]. (**c**) SEM morphology of electrode materials after 100 cycles (upper) and 1000 cycles (lower), revealing progressive microstructural degradation caused by long-term thermal stress. Reproduced from [20].

Figure 1 systematically demonstrates the necessity of lithium-ion battery thermal management from three critical perspectives: cyclic consistency degradation, catastrophic thermal runaway hazards, and irreversible electrode microstructural damage. Figure 1a illustrates the evolution of total resistance and ohmic resistance for parallel-connected battery cells at 80% state of charge over repeated charge–discharge cycles. After 1400 cycles, cells operated at elevated temperatures exhibit a far larger rise in internal resistance than their low-temperature counterparts. This outcome verifies that a temperature gap of merely 5 °C acts as a key engineering threshold triggering accelerated cell inconsistency, which indicates that qualified thermal management requires not only temperature maintenance but also intra-module temperature homogenization. Figure 1b presents the overcharge-triggered thermal runaway characteristics from two complementary perspectives. The representative flame image (upper), captured at the most intense combustion moment during the overcharge experiment, intuitively illustrates the severity of thermal runaway hazards. The synchronous temperature–voltage profiles (lower) quantitatively reveal the catastrophic failure evolution: the battery voltage rises gradually during overcharging but drops abruptly once thermal runaway initiates, while the surface temperature surges sharply to a peak of 935 °C with a violent gas ejection stage lasting approximately 29 s. Together, the visual evidence and quantitative data provide direct baseline references for evaluating the thermal runaway delaying performance of flame-retardant PCM systems discussed in Section 3.1. Figure 1c compares SEM micrographs of electrode surfaces after 100 and 1000 charge–discharge cycles, intuitively showing the progressive growth of surface cracks from minor defects to penetrating fractures. It microscopically reveals the irreversible degradation pathway induced by long-term accumulated thermal stress. Taken together, the three sets of results in Figure 1 lead to a unified core inference: standalone single heat dissipation strategies fail to simultaneously mitigate low-temperature power loss and high-temperature safety hazards. This constitutes the fundamental driving force for introducing PCM technology that integrates thermal buffering and temperature homogenization functions.

Traditional active thermal management solutions are dominated by air cooling and liquid cooling. Air cooling is simple and low-cost but suffers from limited heat dissipation capacity, failing to maintain safe temperatures under discharge rates above 2 C and often causing large temperature gradients across packs [21,22]. Liquid cooling delivers higher cooling efficiency via cold plates or immersion designs, but introduces system complexity, weight penalty and leakage risks [23]. In contrast to these active systems, PCM thermal management technology represents a passive approach that leverages the latent heat of phase transitions to regulate temperature. The working principle of PCMs involves the absorption or release of significant amounts of latent heat during the solid–liquid phase transition, while the temperature of the material remains approximately constant. This characteristic allows PCMs to act as thermal buffers, absorbing excess heat from the battery during high-load conditions and releasing it when the battery temperature drops [24]. The advantages of PCM-based thermal management are supported by substantial research. Operating passively, PCMs require no additional energy consumption, thereby enhancing overall system energy efficiency and reliability [25]. In terms of peak temperature suppression, the latent heat absorption during phase change effectively mitigates battery temperature rise; for instance, an experimental study of graphene skeleton-reinforced composite PCM reported a 13.2 °C reduction in maximum battery temperature under 3 C discharge, along with a 42% improvement in temperature uniformity compared to traditional expanded graphite-based systems [26]. Furthermore, the isothermal nature of the phase transition significantly improves temperature uniformity within the battery pack, with experimental data demonstrating that the maximum temperature difference can be reduced from 8 °C to below 3 °C [25]. PCMs are also well suited to manage the intermittent heat generation characteristic of electric vehicle operation—such as during start-stop, acceleration, and deceleration cycles—by utilizing idle periods to release stored heat and achieve self-regeneration, thereby maintaining battery temperature within a safe range through repeated melting-solidification cycles under dynamic driving conditions [27]. Additionally, in extreme thermal runaway scenarios, PCMs serve as an effective heat sink; overcharge trigger experiments with flame-retardant PCM modules have shown that thermal runaway propagation time is extended by 5.7 times, providing critical windows for safety interventions [28].

**Figure 2:**
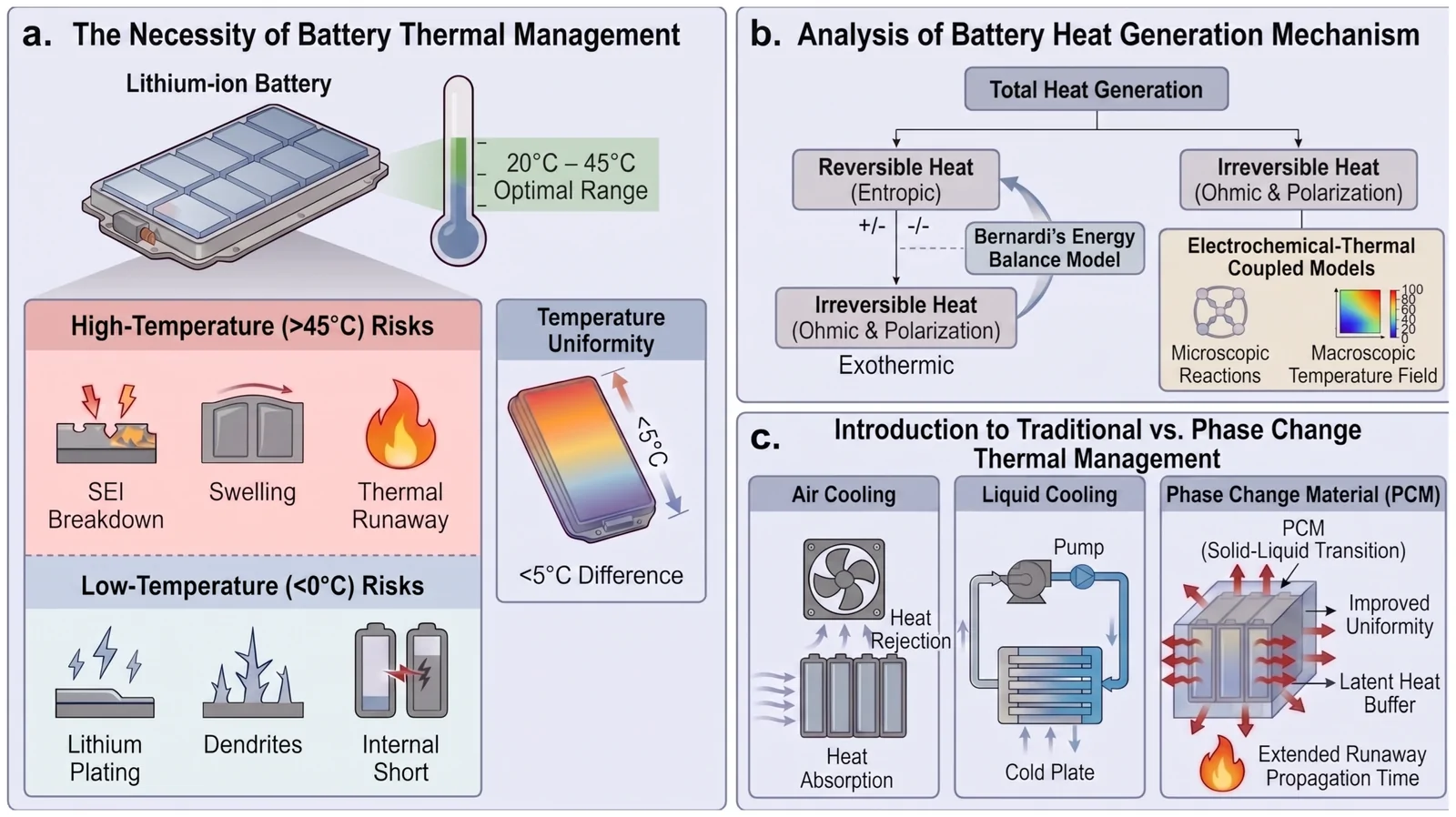
Logical framework of lithium-ion battery thermal management. (**a**) Necessity of battery thermal management and temperature-related risks. (**b**) Analysis of battery heat generation mechanism and theoretical models. (**c**) Comparison of traditional active cooling and PCM-based passive thermal management technologies. Red solid arrows indicate heat-flow direction; black hollow arrows represent cooling medium migration paths; the curved arrow marks the propagation path of thermal runaway.

Despite these distinct advantages, pure passive PCM configurations have inherent operational boundaries. Under sustained high-rate charge–discharge cycles, the PCM may become fully melted and unable to regenerate in time, leading to gradual heat accumulation and loss of sustained temperature control capability. This inherent limitation motivates the development of hybrid thermal management systems that combine PCMs with active cooling technologies to achieve long-term stable performance. The logical framework of battery thermal management, from problem motivation to mechanism analysis and technical solutions, is systematically organized in Figure 2. Figure 2a summarizes the necessity of thermal management, outlining the risks of high- and low-temperature environments, as well as the requirement for inter-cell temperature uniformity, to define the core objectives of thermal regulation. Figure 2b decomposes the battery heat generation mechanism, classifying total heat generation into reversible entropic heat and irreversible ohmic/polarization heat, and introduces the classical theoretical models and simulation methods for thermal behavior prediction. Figure 2c compares three representative thermal management routes—air cooling, liquid cooling, and PCM-based passive cooling—presenting their structural characteristics and core advantages, and highlighting the unique value of PCM technology in temperature uniformity improvement and thermal runaway mitigation.

This review focuses on the application research progress of phase change materials in lithium-ion battery thermal management, beginning with an introduction to the necessity of battery thermal management, an analysis of heat generation mechanisms, and a review of the limitations of traditional technologies to highlight the unique advantages of phase change technology. From the material perspective, the synthesis and modification strategies of PCMs are examined, including thermal conductivity enhancement, phase change temperature regulation, and flame retardancy. From the system perspective, PCM-based thermal management technologies and research progress on hybrid systems combining PCM with other approaches are reviewed. Finally, the full text is summarized and future research directions are discussed. Through this systematic review from the dual dimensions of materials and systems, this work aims to provide a reference for in-depth research on phase change materials in the field of battery thermal management.

## 2. Synthesis Techniques of Phase Change Materials for Battery Thermal Management

### 2.1. Thermal Conductivity Enhancement

Organic phase change materials, such as paraffin and fatty acids, are widely favored for their high latent heat, chemical stability, and non-corrosiveness. However, their inherently low thermal conductivity—typically approximately 0.2–0.3 W/(m·K)—poses a significant bottleneck [29]. This low intrinsic thermal conductivity severely restricts the overall heat transport capability within the battery pack, rather than only affecting local heat transfer processes, leading to thermal accumulation at the cell surface and degraded thermal management performance. Enhancing thermal conductivity is, therefore, a primary focus of material synthesis research. The current mainstream enhancement strategies involve integrating high thermal conductivity additives to form composite phase change materials (CPCMs), aiming to construct a continuous three-dimensional thermal conduction network while minimizing latent heat loss [30].

**Figure 3:**
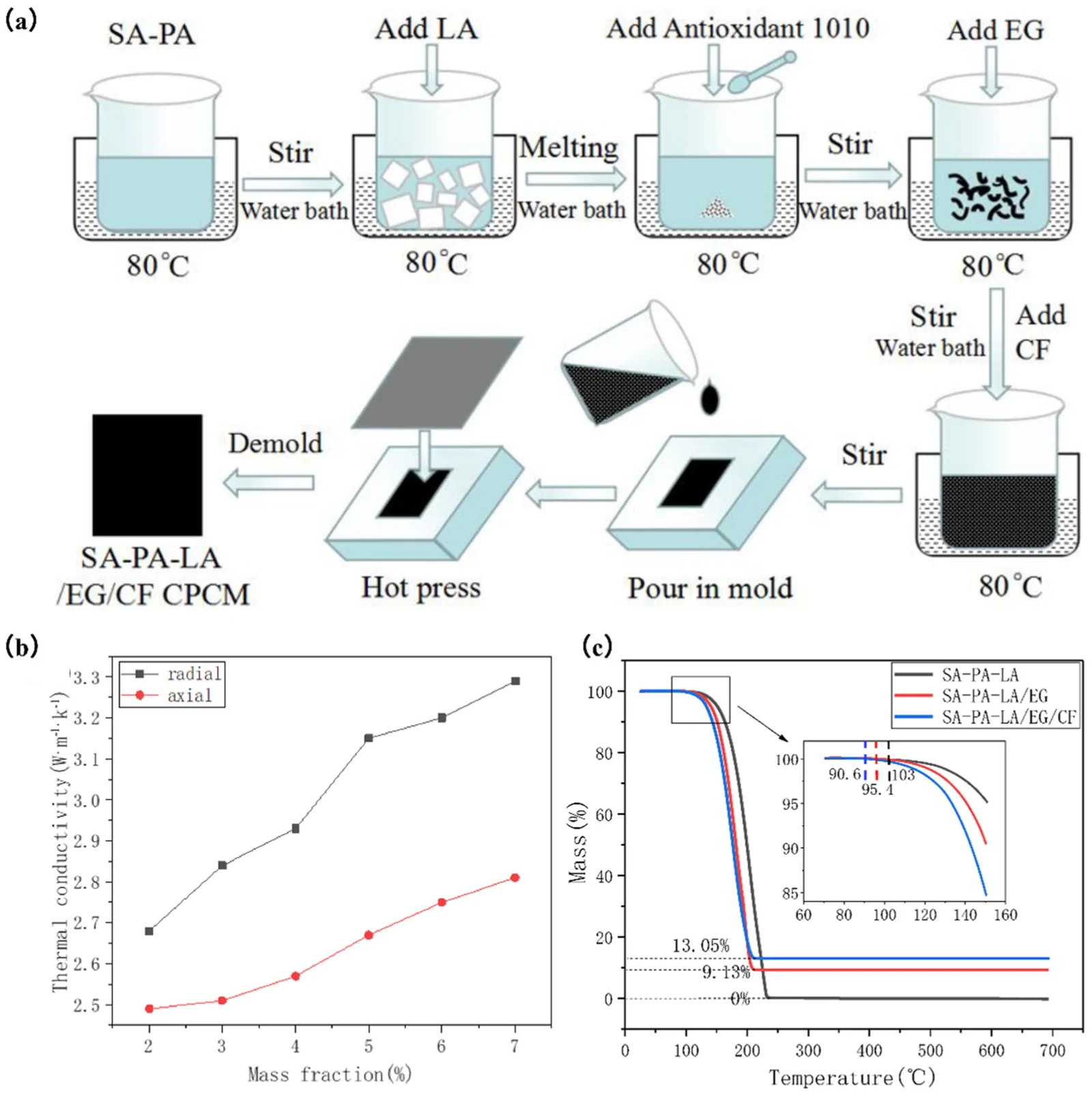
(**a**) Schematic of the melt-blending and hot-pressing fabrication procedure for SA–PA–LA/EG/CF CPCM. (**b**) Anisotropic thermal conductivity of the composite as a function of carbon fiber mass fraction, showing preferential axial heat transport induced by fiber orientation. (**c**) Thermogravimetric analysis curves comparing the thermal stability of SA–PA–LA, SA–PA–LA/EG and SA–PA–LA/EG/CF samples. Reproduced under the CC BY 4.0 license from [31].

To visually demonstrate the typical fabrication route and core thermophysical properties of carbon-reinforced CPCMs, a representative stearic acid–palmitic acid–lauric acid/expanded graphite/carbon fiber (SA–PA–LA/EG/CF) system is presented as a case study in Figure 3 [31]. Taking the ternary fatty acid composite reinforced with expanded graphite and carbon fiber (SA–PA–LA/EG/CF) as a representative case, Figure 3 systematically illustrates the modification logic of composite phase change materials (CPCMs) following a clear logical sequence: fabrication procedure, thermophysical response, and thermal stability verification. Figure 3a elaborates the melt-blending and hot-pressing manufacturing route, which clarifies the encapsulation effect of porous EG skeletons and the loading approach of carbon fiber (CF) fillers. The hot-pressing treatment induces preferred axial alignment of CFs, and this structural characteristic is directly validated by the thermal conductivity data plotted in Figure 3b. Once the CF mass fraction exceeds 4 wt%, the axial thermal conductivity rises far more rapidly than the radial counterpart, which verifies that oriented fibers construct preferential heat transport pathways inside the composite matrix. Finally, the TGA comparison curves presented in Figure 3c confirm the excellent thermal stability of the prepared composites. The initial decomposition temperature of all samples exceeds 90 °C, which is substantially higher than the regular operating temperature window of lithium-ion batteries. In addition, the residual mass of each composite matches the total loading amount of EG and carbon fiber, which demonstrates that only physical adsorption occurs between the fatty acid PCM matrix and carbon fillers without unfavorable chemical reactions that would degrade thermal durability. In summary, Figure 3 reveals a well-defined thermal conductivity optimization route dominated by hot-pressing-induced fiber orientation and corresponding anisotropic heat transport behavior, forming a complete fabrication–structure–property evidence chain to guide the rational design of thermally enhanced CPCMs for battery thermal management.

Carbon-based composites represent a widely adopted approach due to the ultra-high intrinsic thermal conductivity and low density of materials such as expanded graphite, graphene, carbon nanotubes, and carbon fibers. Niu et al. [32] developed a phase-change encapsulation material through electrostatic self-assembly of functionalized expanded graphite and boron nitride, achieving a 200.4% increase in thermal conductivity, which consequently reduced the surface temperature of a simulated battery module by 5.12 °C and 2.41 °C during the final heating stage. Bharathiraja et al. [33] demonstrated that adding graphene nanoplatelets and nano-silica to paraffin could enhance the thermal conductivity of the composite PCM by up to 50% compared to pure paraffin. Zhao et al. [34] prepared a novel flexible PCM with a wide flexibility temperature range, achieving a 503.3% enhancement in thermal conductivity over pure paraffin. Under flexible PCM-based cooling conditions, the single-cell temperature was reduced by 16.9–39.7% compared to natural cooling. Low-dimensional carbon materials such as graphene and carbon nanotubes can construct efficient thermal conduction networks at lower filler loadings, further reducing latent heat loss, and have become a research hotspot in conductivity enhancement [26,35]. Nevertheless, from a manufacturing perspective, high-quality graphene and carbon nanotubes face a prominent cost barrier. As summarized in systematic review research on scalable nano-enhanced phase change materials, low-cost scalable carbon additive manufacturing routes can cut raw material expenditure by 20–35%, proving expanded graphite-reinforced composites deliver optimal cost-performance balance for medium-to-long service battery thermal management scenarios; high-purity graphene and carbon nanotube nanomaterials remain economically uncompetitive for mass industrial deployment under current production conditions [36].

Beyond raw material cost, achieving homogeneous dispersion of nanoscale carbon fillers in organic PCM matrices remains another core bottleneck for mass production. Graphene and carbon nanotubes intrinsically tend to agglomerate via strong van der Waals interactions; inconsistent dispersion in large-batch manufacturing leads to notable performance fluctuations and elevated local interfacial thermal resistance, which undermines the long-term reliability of module-level thermal management. Current scalable fabrication routes for carbon-based composite PCMs mainly include melt blending, in situ polymerization, and prefabricated 3D skeleton impregnation: melt blending is the most industrially compatible process but struggles with uniform dispersion at high filler loadings; in situ polymerization delivers better dispersion homogeneity but increases process complexity and production cycle; prefabricated porous skeleton impregnation, represented by the mature expanded graphite technology, achieves the best balance between performance and scalability, while equivalent graphene/carbon nanotube monoliths are still limited to lab-scale preparation due to prohibitively high fabrication costs [37]. Overall, low-dimensional carbon nanocomposites deliver excellent performance under laboratory conditions, but their full industrial deployment is still constrained by the dual challenges of batch-to-batch consistency and total cost of ownership. For the foreseeable future, expanded graphite remains the mainstream choice for large-volume commercial applications, with graphene/carbon nanotube-based materials reserved for high-end, low-volume scenarios with strict weight and performance requirements.

**Figure 4:**
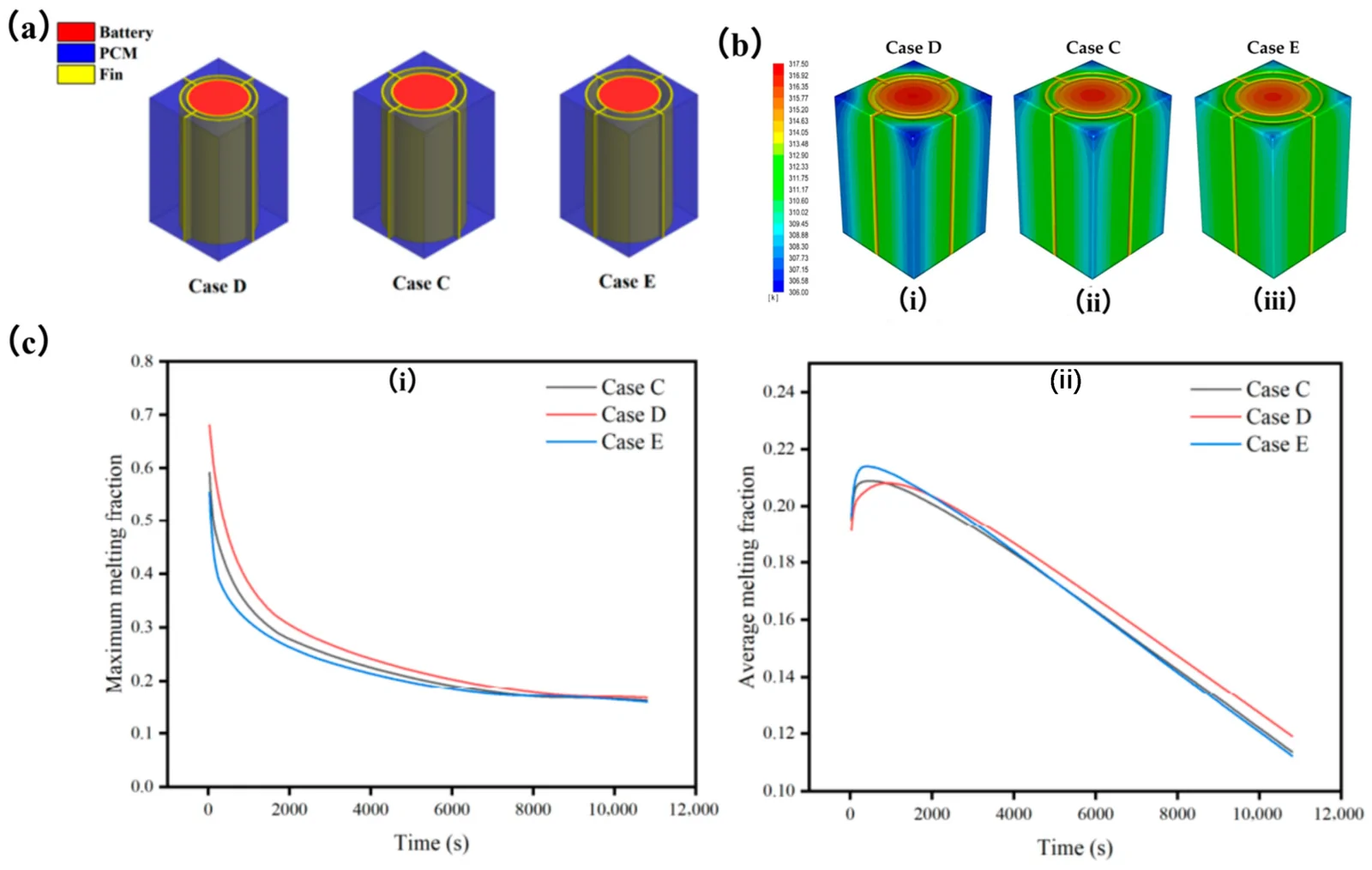
(**a**) 3D geometric models of three representative PCM-fin coupled configurations (Case D, C, E) with annular fin spacings of 2 mm (Case D), baseline (Case C), and 4 mm (Case E), respectively. (**b**) Steady-state temperature contours after 5 C discharge for Case D, C and E. (**c**) Time-dependent evolution of peak battery temperature and inter-cell temperature difference during the post-discharge solidification stage, directly reflecting the dual core objectives of thermal management (temperature suppression and uniformity). Reproduced under the CC BY 4.0 license from [42].

Metal-based composites also offer significant improvements. Metal foams, grids, and fibers provide a three-dimensional continuous thermal conduction skeleton [38,39]. Li et al. [40] proposed a novel hybrid battery thermal management strategy combining metal foam/paraffin composites with a thermal bridge, significantly enhancing thermal conductivity and reducing the maximum battery temperature and temperature difference by 44.27 °C and 11.1 °C, respectively. Yetik et al. [41] added 5 wt% aluminum powder to paraffin, achieving a thermal conductivity of 0.2612 W/(m·K) for the Triply Periodic Minimal Surface-Paraffin Wax composite at room temperature, a 68% increase over pure paraffin. In addition to particle and porous skeleton filler modification, the design of integrated aluminum fin networks is another effective technical route to break the low intrinsic heat transport bottleneck of pure organic PCMs. A set of multi-group fin layout models and their corresponding steady-state temperature, liquid fraction and solidification-stage quantitative simulation results are summarized in Figure 4, reproduced under the CC BY 4.0 license from reference [42]. Beyond filler-based thermal conductivity enhancement, structural optimization represents another critical strategy to address the intrinsic low heat transport bottleneck of neat organic PCMs. Taking a cylindrical 26,650 battery module as the carrier, Figure 4 investigates the effect of annular fin spacing on thermal performance using three representative configurations (Case D, C, E).

As shown in Figure 4a, all layouts share vertical longitudinal fins with additional annular rings, while the spacing varies from 2 mm (Case D) to a medium baseline (Case C) and 4 mm (Case E). The steady-state temperature nephograms in Figure 4b reveal that, as the spacing increases from 2 mm to 4 mm, the high-temperature hot zones adjacent to cell surfaces shrink significantly and the overall temperature gradient within the PCM matrix is markedly alleviated. Quantitative comparisons are consolidated in Figure 4c, which plots the time-dependent evolution of peak battery temperature and inter-cell temperature difference—the two metrics directly corresponding to the core objectives of thermal management. During the 1800 s post-discharge cooling stage, all cases exhibit rapid initial drops in both parameters. However, Case E consistently maintains the lowest peak temperature and the smallest temperature difference, with the steady-state ΔT stabilized below 0.2 K. This confirms that, under the given geometric constraints, the 4 mm spacing achieves the optimal balance between heat conduction enhancement and full utilization of latent heat, providing explicit design guidance for fin-equipped PCM modules. This structural optimization echoes the trade-off principle discussed in the preceding section: whether through filler doping or skeleton design, the core target is the synergistic matching of heat transport and heat storage capacities, rather than the one-sided pursuit of ultra-high thermal conductivity.

**Figure 5:**
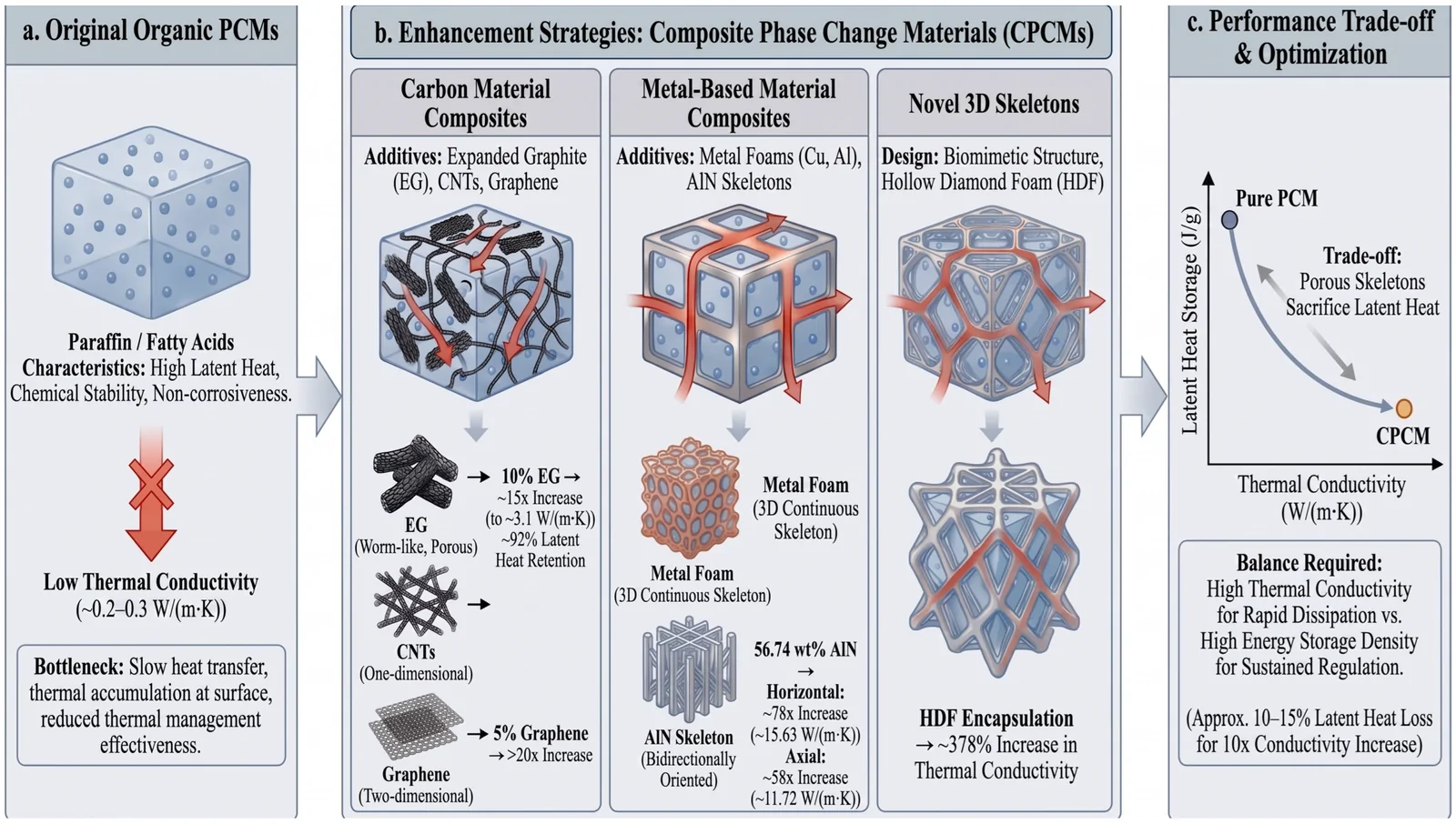
Schematic of different thermal conductivity enhancement strategies for organic PCMs, showing different filler structures to build thermal conduction networks. Black solid arrows represent thermal conduction pathways; red arrows indicate performance-trend directions.

It is important to note that the introduction of high-thermal-conductivity fillers often comes at the cost of some latent heat capacity. A 2024 meta-analysis of over 200 composite PCM studies validates that a 10-fold thermal conductivity enhancement typically brings 10–15% latent heat loss for randomly distributed fillers, while oriented structured skeletons can reduce this loss to below 7% through higher filler utilization efficiency [30]. Therefore, the design of CPCMs requires a careful balance to achieve both high thermal conductivity for rapid heat dissipation and high energy storage density for sustained thermal regulation [43]. The core research objective is to find the optimal balance between high thermal conductivity and high latent heat through filler structure design, interfacial modification, and topological optimization [44,45]. To help compare the characteristics of different thermal conductivity enhancement strategies, we summarize their performance, cost and applicable scenarios in the following Table 1. The schematic illustration of these different enhancement strategies and their corresponding thermal conduction network structures is provided in Figure 5. This figure systematically shows the schematic diagram of different thermal conductivity enhancement strategies for organic PCMs, clearly presenting how different high thermal conductivity fillers build continuous thermal conduction networks inside the PCM. For example, carbon-based fillers, metal-based fillers and other different addition methods, how to build three-dimensional heat transfer paths to solve the problem of low intrinsic thermal conductivity of organic PCMs, and also intuitively compare the influence of different filler structures on the construction of thermal conduction networks, helping to understand the action mechanism of different thermal conductivity enhancement schemes.

### 2.2. Phase Change Temperature Regulation

The phase change temperature of a PCM must match the optimal operating range of the battery (typically 20–45 °C) to be effective. Different climate zones and operating conditions impose varying requirements, necessitating controllable and wide-range tuning of the PCM’s phase change temperature. Current strategies for phase change temperature regulation are primarily classified into chemical synthesis modification and physical blending modification [46]. Geng et al. [47] synthesized seven novel ester-based PCMs by esterifying lauric acid with seven different alcohols. This chemical method significantly broadened the phase change temperature range of the materials, covering a melting point range from −8.99 °C to 46.60 °C, which is a 203.18% expansion compared to the raw alcohol materials. This approach enables precise tuning of the phase change temperature through molecular design to suit different thermal management needs.

Physical blending, particularly the construction of binary or multi-component eutectic systems, offers a straightforward method for continuous phase change temperature adjustment over a wide range with minimal latent heat loss, making it highly promising for engineering applications. Chen et al. [48] developed a novel multifunctional flame-retardant solid-solid PCM through a chemical modification process. Using polyethylene glycol 2000 as the phase change matrix, and through the synergistic regulation of boric acid, phosphorus pentoxide, and expanded graphite, they achieved continuous tunability of phase change enthalpy and temperature within the range of 5–60 °C. This material also demonstrated excellent thermal stability over 1000 cycles, along with superior mechanical stability and flame retardancy, making it ideally suited for the full-operating-condition thermal management of power batteries. Additionally, the nanoconfinement effect of porous materials can be used to regulate the phase change behavior of PCMs. For example, paraffin confined in SBA-15 mesoporous silica achieves a 3–8 °C reduction in melting point via pore size control, while metal–organic framework derived porous hosts enable finer tuning of transition temperature through tailored pore structures and host–guest intermolecular interactions. However, the range of temperature adjustment is generally limited to within 10 °C, and the complex preparation processes coupled with high precursor costs hinder large-scale industrial application [49]. Overall, achieving precise control of phase change temperature through molecular design and enabling wide-range adaptation through eutectic systems are the two core development directions in phase change temperature modification [50].

### 2.3. Flame-Retardant and Multifunctional Phase Change Materials

Safety is a paramount concern for battery applications. Traditional organic PCMs (paraffin, fatty acids) are hydrocarbon compounds with significant flammability. In battery thermal runaway scenarios, leakage of high-temperature internal substances can easily ignite the PCM, creating a vicious cycle of “thermal runaway—material combustion—further heat release” and exacerbating safety risks [51]. Consequently, flame retardant modification has become a crucial research direction to enhance PCM safety, alongside multifunctional integration including flexibility, shape stability, and electrical insulation.

Current flame retardant modification strategies are mainly divided into intrinsic flame-retardant molecular design and additive flame retardant compounding. Intrinsic flame retardant modification introduces flame-retardant elements such as silicon, phosphorus, and nitrogen into the PCM molecular chain, endowing the material with inherent flame retardancy. This approach offers advantages such as high efficiency, minimal impact on latent heat, and good long-term stability. Zheng et al. [52] synthesized a novel silane-modified 1-hexadecanol intrinsic flame-retardant PCM via a nucleophilic addition reaction between 3-isocyanatopropyltrimethoxysilane and 1-hexadecanol. The material exhibited a high latent heat of 115.1 J/g, with a peak phase change temperature in the ideal range of 30–45 °C, while the introduction of the silane group significantly enhanced its flame retardancy, meeting both the temperature control and safety requirements for power battery thermal management.

Additive flame retardant modification, which involves incorporating intumescent flame retardants or inorganic flame-retardant fillers into the PCM matrix to build a synergistic flame-retardant system, is the most widely used method due to its simple process, low cost, and strong adaptability [53,54]. Wang et al. [55] prepared a composite PCM based on sodium sulfate decahydrate with functional flame-retardant fillers through a multi-step process. This material exhibited high thermal conductivity, good cyclic stability, and excellent flame retardancy, remaining non-flammable even at high temperatures of 350 °C, as verified by fire resistance tests. As a core quantitative indicator of flame-retardant performance, the limiting oxygen index of optimized synergistic flame-retardant PCM systems can reach over 42%, achieving the self-extinguishing level and effectively blocking the combustion chain reaction during thermal runaway [56].

**Figure 6:**
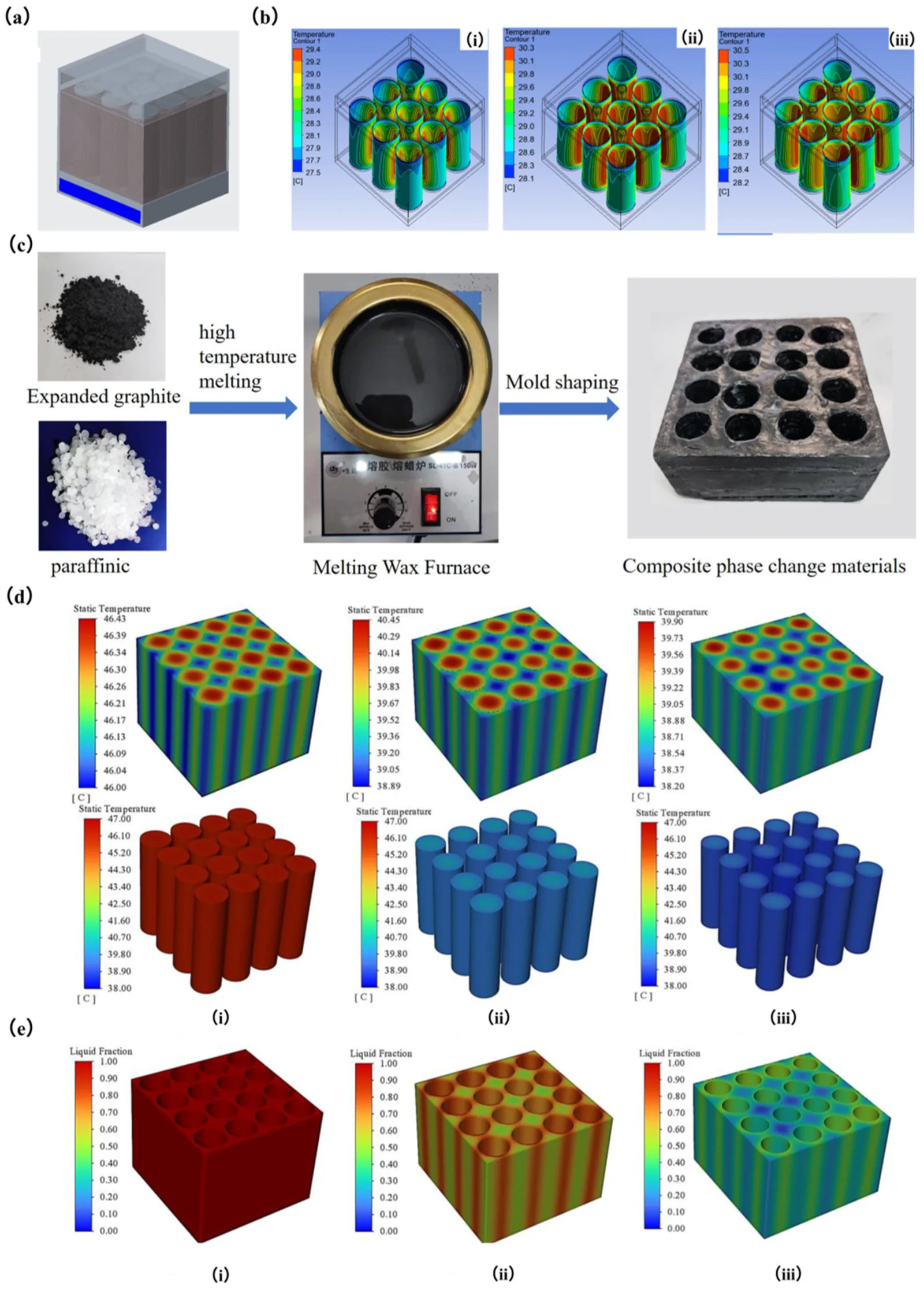
(**a**) Isometric view of the proposed hybrid thermal management concept, showing the cylindrical Li-ion cells, PCM enclosure, vertical liquid channels, and top air duct. Reproduced from [60] (Figure 1a). (**b**) Temperature contours at discharge rates of 1 C, 3 C and 5 C, illustrating the cooling performance of the hybrid design across representative operating conditions. Reproduced from [60]. (**c**) Melt-blending fabrication process of expanded graphite-paraffin composite PCM, illustrating the scalable preparation route from material to system integration. Reproduced from [61]. (**d**) Temperature distribution of the CPCM module with different PCM filling thicknesses (2 mm, 4 mm, and 6 mm). Reproduced from [61]. (**e**) Corresponding liquid fraction distribution of the CPCM module with different PCM filling thicknesses (2 mm, 4 mm, and 6 mm). Reproduced from [61]. Note: The Chinese mark in (**c**) comes from the reproduced original source figure.

Beyond flame retardancy, flexibility modification is another important direction in multifunctional research. Power batteries in vehicles are subject to long-term vibration, which can cause interfacial detachment and structural damage in rigid PCMs, leading to a significant increase in interfacial thermal contact resistance and a decline in thermal management performance. Flexible composite PCMs, leveraging the synergistic effect of a polymer matrix and a porous scaffold, can maintain excellent interfacial contact, reduce contact resistance, and enhance vibration resistance, making them suitable for complex automotive service conditions [57,58]. Currently, the development of multifunctional integrated PCMs that combine high thermal conductivity, high latent heat, intrinsic flame retardancy, shape stability, and flexible adaptability represents a frontier research direction in this field [59]. Covering the complete research chain from material synthesis to system integration, Figure 6 illustrates typical configurations and characterization results of CPCM hybrid thermal management systems following a progressive logical sequence: fabrication fundamentals, structural design, performance verification, and parametric optimization.

First, Figure 6c demonstrates the melt-blending manufacturing procedure for expanded graphite/paraffin composite PCMs. This facile technique imposes low equipment requirements and is highly compatible with large-scale industrial production, laying a feasible foundation for practical engineering applications. Supported by this mature material preparation route, the three-in-one hybrid thermal management architecture is visualized in the 3D schematic of Figure 6a, which integrates passive thermal buffering from CPCMs, sensible heat removal via internal liquid channels, and supplementary heat regeneration through top forced airflow. This explicitly reflects that material fabrication is developed to serve system integration, rather than functioning as an independent standalone component.

On the basis of the above structural layout, Figure 6b verifies the temperature control capacity of the hybrid scheme under three representative discharge rates (1 C, 3 C and 5 C). Even at the severe 5 C high-rate operating condition, the module peak temperature is stabilized at approximately 30 °C, which fully validates the effectiveness of the proposed integrated design. Furthermore, Figure 6d,e compare the module temperature distribution and corresponding PCM liquid fraction profiles under three filling thicknesses (2 mm, 4 mm and 6 mm). When the PCM thickness is set to 2 mm, the phase change material reaches a full liquid fraction of 100% and loses its temperature regulation capability completely. Although the 6 mm configuration delivers superior cooling performance, it increases the overall module volume by around 15% and sacrifices pack-level energy density. In contrast, the 4 mm filling scheme achieves a balanced solution: it restricts the liquid fraction below 80 °C and limits inter-cell temperature differences to less than 5 °C while maximizing volumetric efficiency. This parametric trade-off analysis addresses the practical engineering question of optimal PCM filling thickness and forms a complete closed-loop design logic spanning composite material development to full module system deployment.

Overall, current material modification strategies have largely overcome the intrinsic defects of organic PCMs, but core trade-offs remain unresolved. For example, high thermal conductivity enhancement is almost always accompanied by latent heat loss, and multifunctional integration often dilutes the energy storage density of the matrix. As systematically compared in Table 1 and Figure 5, different enhancement strategies show distinct performance-cost trade-offs, and there is still no universal one-size-fits-all solution. Balancing multiple performance parameters with a simple, scalable fabrication process remains the biggest challenge for material-level development.

### 2.4. Cyclic Stability and Long-Term Aging Mechanisms

For electric vehicle applications, PCM-based thermal management systems are required to maintain reliable performance over thousands of melting-solidification cycles across a service life of 8–10 years. While many modified PCMs have been reported to retain stable thermal properties after 1000 cycles, the underlying physical and chemical degradation mechanisms during long-term cycling remain insufficiently discussed, which poses a major barrier to practical industrial deployment. The primary aging pathways of organic PCM composites include phase segregation, evaporative leakage, and matrix oxidation.

Phase segregation is the most prevalent degradation mode for composite and eutectic PCMs. For binary or multi-component eutectic systems, repeated phase transitions can gradually cause component separation and non-uniform crystallization, leading to drift in phase change temperature and attenuation of latent heat. For filler-reinforced composite PCMs, long-term cyclic volume expansion and contraction can induce interfacial debonding between the PCM matrix and thermally conductive fillers, disrupting the continuous thermal conduction network and increasing interfacial thermal resistance. Long-term cycle tests demonstrate that conventional paraffin/expanded graphite composites suffer a 14–29% decline in effective thermal conductivity after 10,000 thermal cycles, alongside latent heat attenuation of 3.6–8.9% driven by interfacial debonding and local phase segregation [62].

Evaporative loss and leakage constitute one of the dominant degradation pathways for unencapsulated organic PCM composites exposed to prolonged high-temperature cycling. Within semi-sealed battery module architectures, molten paraffin substrates gradually undergo surface volatilization and interfacial migration during repeated melting-solidification transitions, leading to progressive attenuation of usable latent heat capacity over long service durations. Recurring volumetric expansion and shrinkage exacerbates PCM extrusion through assembly clearances, gradually eroding sustained thermal regulation performance. A dual microcapsule plus rigid ceramic skeleton encapsulation architecture validated in recent high-temperature PCM research delivers robust suppression of volatilization, matrix outflow and structural rupture across 5000 heating-cooling cycles; the composite only achieves a minimal 3.3% oxidative mass gain and retains over 80% initial latent enthalpy after long-duration cycling, providing a viable structural strategy to mitigate evaporative and leakage-driven degradation [63].

Thermal oxidation and chemical degradation occur under prolonged high-temperature and aerobic conditions. Hydrocarbon-based organic PCMs such as paraffin and fatty acids can undergo oxidative chain scission and functional group evolution after long-term exposure to elevated temperatures, resulting in reduced chemical stability and shifted phase transition behavior. In addition, oxidative aging at the filler-matrix interface can weaken interfacial bonding, further amplifying performance degradation. For flame-retardant modified systems, the migration and precipitation of small-molecule flame retardants during cycling can also gradually weaken the flame retardant effect over time [64].

To address these aging issues, several optimization strategies have been developed. Form-stable structural design based on cross-linked polymer networks or three-dimensional porous skeletons can effectively suppress leakage and phase segregation by physically confining the PCM matrix. Microencapsulation technology further isolates the PCM from the external environment, reducing both evaporative loss and oxidation risk. Additionally, the incorporation of antioxidants and interfacial modifiers can improve the oxidation resistance of the matrix and enhance interfacial bonding strength, extending cycle life.

Overall, current cyclic stability studies are mostly limited to 1000–2000 cycles under laboratory conditions, and long-term aging data under actual vehicle operating conditions (temperature fluctuation, vibration, multi-field coupling) are still scarce. Systematically revealing the multi-factor coupled aging mechanism and establishing accelerated life evaluation methods are important prerequisites for promoting the large-scale application of PCMs in automotive thermal management.

### 2.5. Environmental Impact, Recyclability and Sustainable Alternatives

With the approaching large-scale deployment of PCM-based battery thermal management systems in electric vehicles, the full-life-cycle environmental impact and end-of-life disposal of these materials have emerged as critical concerns for industrial sustainability. This is particularly true for nanomaterial-reinforced and flame-retardant composite PCMs, whose ecological fate after battery retirement remains largely underexplored. From a life cycle perspective, the environmental footprint of PCM systems spans raw material production, manufacturing, long-term service, and final disposal, requiring systematic trade-off analysis between thermal performance and ecological cost.

#### 2.5.1. Life Cycle Assessment of Nanocomposite PCMs

Life cycle assessment results indicate that petroleum-derived organic PCMs such as paraffin already carry notable carbon emissions from fossil feedstock extraction and refining. The introduction of nanoscale functional fillers further amplifies the environmental burden: the redox exfoliation route for graphene production delivers a cradle-to-gate carbon footprint up to 256.5 kg CO_2_ eq/kg, while the production of conventional natural graphite emits less than 10 kg CO_2_ eq/kg, representing a difference of more than one order of magnitude [65]. For flame-retardant formulations, halogenated additives pose persistent organic pollutant risks during both production and end-of-life incineration, while phosphorus-based alternatives, though comparatively safer, still carry potential eutrophication risks if released into aquatic environments. Recent comprehensive review studies indicate that scalable low-cost carbon additive routes can cut raw material costs by 20–35%, which proves that expanded graphite-reinforced composites deliver the optimal balance between thermal performance and overall comprehensive performance for medium-to-long service scenarios; by contrast, high-end nanocarbon fillers only display competitive merits under extreme lightweight and high-efficiency operating conditions [36].

#### 2.5.2. End-of-Life Fate and Recycling Barriers of Nanocomposite PCMs

At the end of battery service life, nanomaterial-containing PCMs currently lack standardized recycling pathways and are mostly disposed of alongside battery packs via landfill or incineration. In landfill scenarios, unbound graphene, carbon nanotubes, and nano-sized flame-retardant particles may leach into soil and groundwater over time, posing potential ecotoxicological risks to soil microorganisms and aquatic ecosystems. Under incineration disposal, the organic PCM matrix generates greenhouse gas and toxic volatile emissions, while nanoscale fillers become enriched in fly ash, creating secondary contamination risks.

Technically, the recycling of nanocomposite PCMs faces two core bottlenecks. First, PCMs are tightly bonded to battery cells, metal housings, and heat dissipation structures during module integration, making clean, non-destructive separation extremely difficult under automated disassembly. Second, the recovery of functional nanofillers faces performance degradation challenges: melt recovery and solvent extraction are the two most studied methods, with melt separation achieving over 90% matrix recovery rate but severely damaging the constructed 3D thermal conduction network; solvent extraction can retain filler morphology to a greater extent but requires large amounts of organic reagents, increasing secondary pollution and recycling costs. To date, closed-loop recycling of nanoscale fillers from waste nanocomposites remains limited to laboratory verification, with no mature industrial-scale process available. Relevant research by Rezaei & Prince further points out that although physical, thermal and chemical recycling routes have been investigated, specific challenges including inhibiting nanoparticle agglomeration and preserving original surface chemical properties are still unresolved [66].

#### 2.5.3. Bio-Based PCMs as Sustainable Development Pathways

Bio-derived phase change materials represent a promising eco-friendly alternative to petroleum-based systems, aligning with the global trend toward low-carbon energy storage technologies. Typical bio-based PCM matrices include plant-derived fatty acids, biowaxes, and sugar alcohols, which are sourced from renewable biomass, exhibit good biodegradability, and can reduce life-cycle carbon emissions by 40–60% compared to paraffin-based equivalents. As validated in driving-cycle experiments by Sharma, bio-based PCMs reinforced with walnut shell-derived biocarbon and reduced graphene oxide deliver a maximum temperature reduction of 12.8 °C for power batteries and maintain stable thermal performance over 490 continuous charge–discharge cycles, demonstrating great practical potential for vehicle thermal management [67].

In terms of reinforcing fillers, biomass-derived porous carbon, cellulose nanofibers, and chitin-based nanomaterials can replace traditional graphite and metal fillers, further improving the full biodegradability of the composite system. Recent advances show that nanocellulose-reinforced bio-based composite PCMs can achieve thermal conductivity enhancement ratios comparable to expanded graphite systems, while maintaining full compostability at the end of service life. Nevertheless, bio-based PCMs still face bottlenecks such as relatively low latent heat per unit volume, poorer long-term oxidation resistance, and higher raw material costs at current production scales, which restrict their large-scale commercial application.

Overall, sustainable design covering the full life cycle of materials has become an indispensable direction for next-generation PCM development. Future research needs to establish standardized life cycle assessment evaluation frameworks for nanocomposite PCMs, develop low-cost, high-efficiency filler recovery processes, and further optimize the performance-cost ratio of bio-based systems, to achieve a balance between thermal management performance and environmental friendliness.

## 3. Thermal Management Performance of Phase Change Materials

### 3.1. Thermal Management Technology Based on Phase Change Materials

The working principle of pure passive PCM-based thermal management involves integrating PCM with battery cells, typically through encapsulation, filling, or potting [68,69,70]. The heat generated during battery operation is transferred to the PCM; when the temperature reaches the PCM’s melting point, it absorbs a large amount of latent heat through solid–liquid phase change, suppressing the peak temperature rise. When heat generation decreases and the temperature drops, the PCM solidifies and releases the stored heat, acting as a thermal buffer. Simultaneously, its high thermal conductivity helps disperse heat evenly within the battery pack, ensuring temperature consistency among cells. This system requires no auxiliary power components, offering the simplest structure and highest reliability. Its core performance is evaluated in terms of cooling effect, temperature uniformity, and thermal runaway delay.

In terms of cooling effect, composite PCMs effectively suppress battery temperature rise due to their high latent heat and high thermal conductivity. Numerous studies have validated this cooling efficacy. Numerical simulation studies indicate that n-octadecane can reduce the maximum temperature of a prismatic lithium battery by 15.7 °C under 2 C discharge conditions, while eicosane can reduce it by 12.2 °C [71,72]. Experimental studies have confirmed that carbon-based/paraffin CPCMs can maintain the operating temperature of lithium battery packs consistently below 50 °C at a 3.75 C discharge rate [26,73,74].

Regarding temperature uniformity, PCMs leverage the isothermal nature of the phase change process and the heat-dispersing capability of high-conductivity networks to significantly improve temperature uniformity within the battery pack, strictly controlling inter-cell temperature differences within safe thresholds. Bian et al. [75] designed a thermal management system incorporating heat pipes and PCMs, which effectively controlled battery pack temperature under self-defined dynamic driving cycles covering constant-speed cruising, uphill climbing, and 120 km/h high-speed operation. The system limited the total temperature rise to below 5 °C and maintained the temperature difference within 2 °C under high-speed conditions. Keyhani-Asl et al. [76] further confirmed that a composite PCM thermal management system enhanced with copper foam could maintain the maximum temperature difference within a battery pack below 1 °C, even at a high discharge rate of 5 C, demonstrating superior temperature uniformity compared to traditional systems.

For thermal runaway delay and protection, composite PCMs can absorb a large amount of sudden heat in the early stages of thermal runaway due to their latent heat and flame-retardant properties, interrupting the chain propagation path and significantly delaying its initiation and spread [77]. Beyond organic-matrix PCMs, nano-encapsulated inorganic PCMs and salt hydrate-based composites have also been verified to effectively inhibit thermal runaway propagation via the synergy of latent heat absorption and thermochemical reactions [78,79]. For instance, research by Shen et al. utilized paraffin wax to delay the onset of thermal runaway by over 10 min under thermal abuse conditions [80]. In overcharge trigger experiments, battery modules using flame-retardant PCM exhibited a 2.5-fold extension in thermal runaway propagation time [56,81,82]. By combining latent heat absorption with flame-retardant properties, these materials achieve a dual protection mechanism of “temperature control + fire suppression,” significantly enhancing the intrinsic safety of battery systems [83,84].

Based on extensive research findings, an ideal PCM for battery thermal management should satisfy the following core criteria [85]: (1) Phase change temperature aligned with the battery’s optimal operating range (20–45 °C); (2) High latent heat capacity (>150 J/g); (3) High thermal conductivity (>1 W/(m·K)); (4) Small volume change during phase transition. A volume expansion ratio below 10% is widely accepted as a general qualifying threshold for PCM module integration, while a stricter limit of <5% is typically specified for organic PCMs in tightly packed commercial battery packs with tight dimensional tolerances, as excessive volume expansion can degrade interfacial thermal contact, increase leakage risk, and induce structural fatigue over long-term cycling [29]; (5) Good chemical and cyclic stability; (6) Excellent flame retardancy and electrical insulation properties.

**Figure 7:**
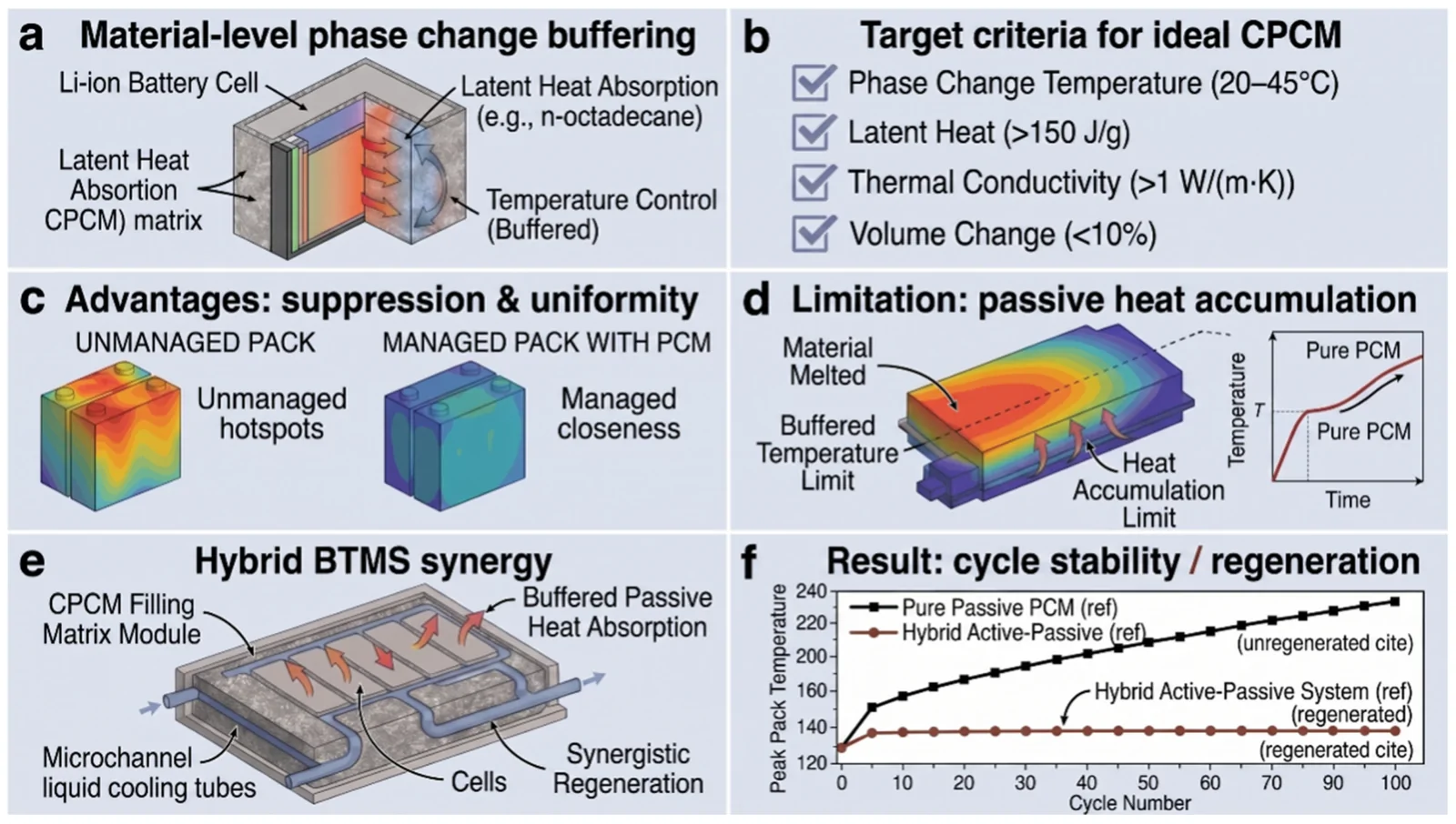
Performance evaluation of phase change material-based lithium-ion battery thermal management systems. (**a**) Mechanism of material-level phase change buffering; (**b**) Target criteria for ideal CPCMs. (**c**) Advantages in peak temperature suppression and inter-cell temperature uniformity. (**d**) Core limitation of heat accumulation in pure passive PCM systems. (**e**) Structural synergy of hybrid active-passive battery thermal management systems. (**f**) Cycle stability and thermal regeneration performance comparison. Note: In subgraphs (**a**,**c**,**d**), different colors indicate temperature distribution. Red represents high-temperature regions, while blue represents low-temperature regions of the battery pack.

Despite these advantages, a key bottleneck remains for pure passive PCM systems: under extreme conditions such as continuous high-rate charge/discharge, if the PCM fully melts and cannot be regenerated in time, it loses its sustained temperature control capability, leading to heat accumulation. Therefore, coupling with active cooling technologies to form hybrid thermal management systems is essential to address this limitation [86]. A comprehensive performance evaluation of PCM-based battery thermal management systems, covering both the advantages of passive cooling and the limitations of pure PCM systems, is summarized in Figure 7. This figure conducts a comprehensive performance evaluation of PCM-based battery thermal management systems from multiple dimensions. Figure 7a explains the mechanism of phase change buffering at the material level, showing how PCM achieves temperature stability by absorbing heat through solid–liquid phase change. Figure 7b clarifies the target performance indicators of ideal CPCMs, providing a direction for the optimal design of materials. Figure 7c intuitively compares the peak temperature and temperature difference of batteries with and without PCM, highlighting the significant advantages of PCM in suppressing peak temperature and improving temperature uniformity. Figure 7d points out the core limitation of pure passive PCM system, that is, the heat accumulation problem under continuous high rate operation, explaining why hybrid thermal management system is needed. Figure 7e shows the structure synergy mechanism of the hybrid active-passive battery thermal management system, explaining how PCM and active cooling complement each other. Figure 7f compares the cycle stability and thermal regeneration performance of different systems, proving that the hybrid system can achieve long-term stable thermal management effect.

### 3.2. Thermal Management Technology Combining Phase Change Materials with Other Technologies

The core design philosophy of hybrid thermal management systems is to combine the passive temperature control and uniformity advantages of PCMs with the sustained heat dissipation capability of active cooling technologies, creating a synergistic “passive control + active removal” system [87]. This approach leverages PCMs to suppress peak temperatures and ensure uniformity, while active cooling removes stored heat from the PCM, promoting its regeneration for long-term, stable operation across all operating conditions [88,89]. This represents the mainstream technical route that balances performance with practicality. Currently, the most widely studied hybrid systems include PCM-air cooling, PCM-liquid cooling, PCM-heat pipe, and full-temperature-range systems coupling PCM with heating [90].

PCM-air cooling hybrid systems are the simplest and lowest-cost hybrid solution. Their core principle is to use forced convection air cooling to remove heat absorbed and stored by the PCM, facilitating its solidification and regeneration. This addresses the heat accumulation issue of standalone PCM systems while compensating for the poor heat dissipation and temperature uniformity on the leeward side in traditional air cooling systems. He et al. [91] proposed a novel PCM-air cooling hybrid battery thermal management system, placing PCM in a semi-elliptical arrangement on the leeward side of the batteries. This configuration effectively absorbs heat on the leeward side where traditional air cooling is less effective, significantly improving temperature uniformity and overall thermal management performance, maintaining battery temperature within the ideal range even under high-rate discharge conditions.

**Figure 8:**
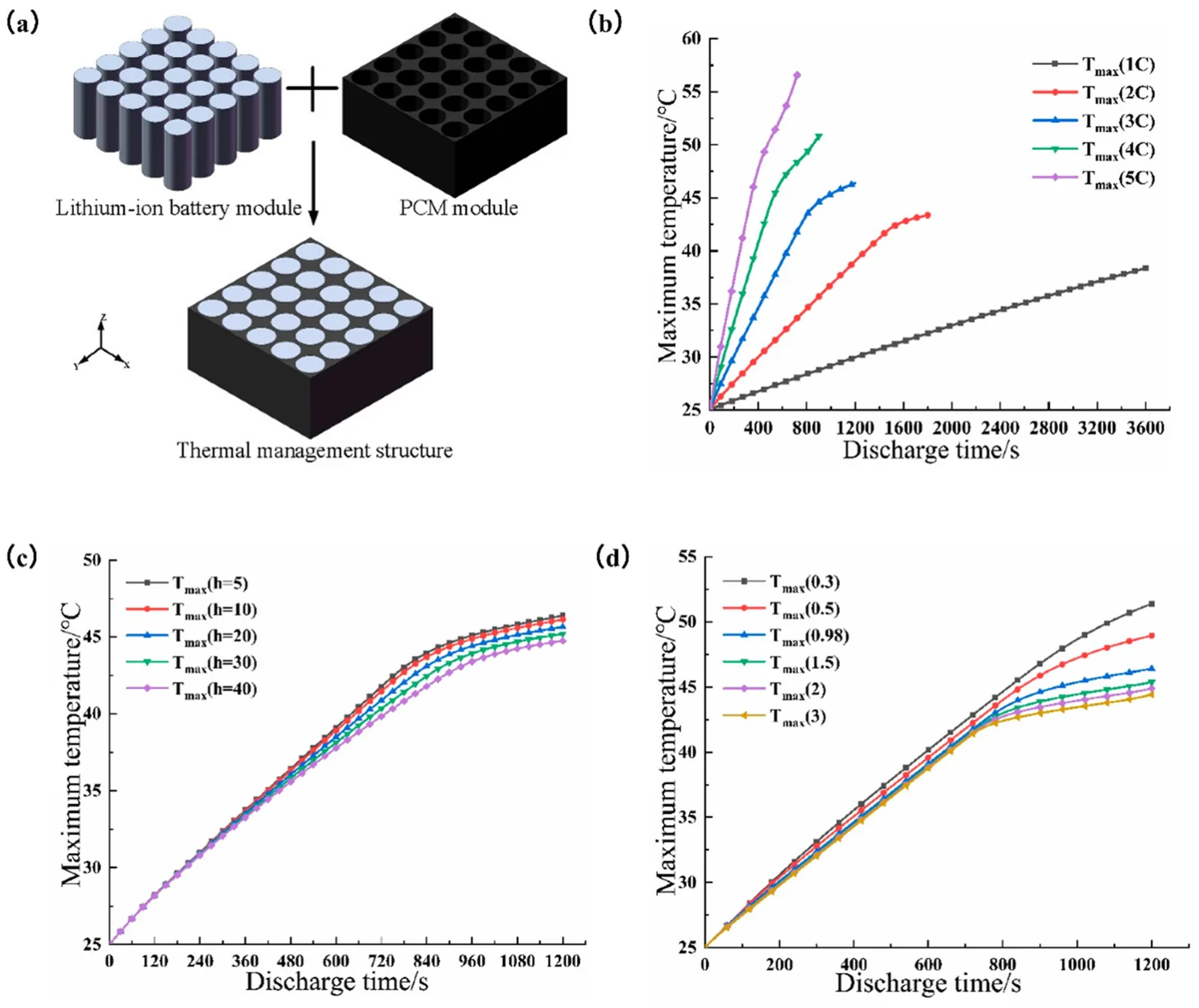
(**a**) Three-dimensional structural model of the 5 × 5 cylindrical lithium-ion battery module encapsulated with SAT-AC composite PCM. (**b**) Evolution of maximum battery temperature at discharge rates of 1 C to 5 C. (**c**) Maximum temperature curves under different external air convective heat transfer coefficients. (**d**) Maximum temperature profiles with varying radial thermal conductivity of battery cells under 3 C discharge. Reproduced under the CC BY 4.0 license from [94].

PCM-liquid cooling hybrid systems combine the efficient and sustained cooling capacity of liquid cooling with the superior temperature uniformity and peak-shaving capabilities of PCMs. This approach currently offers the best cooling performance among hybrid solutions, making it particularly suitable for high-rate charge/discharge and high-energy-density power batteries. Nevertheless, the integration of both PCM matrix and liquid cooling components introduces notable volumetric and gravimetric penalties at the pack level, trading off system energy density for enhanced thermal performance. Zhou et al. [92] investigated an innovative hybrid battery thermal management system combining PCM with a honeycomb microchannel liquid cooling plate. This system maintained a maximum battery temperature (Tmax) of 29.1 °C and a temperature difference (ΔT) of 3.1 °C under standard operating conditions. Wang et al. [93] proposed a novel hybrid cooling system incorporating PCM and a wavy microchannel cooling plate. Their findings indicated that the hybrid system using PCM and microchannel cooling plates achieved significantly lower battery temperatures compared to standalone PCM or air cooling systems. To systematically analyze the performance limitations of standalone CPCM passive thermal management and quantify the influence of three core design parameters (discharge rate, cell radial thermal conductivity, external convection coefficient), a multi-dimensional parametric simulation based on a 5 × 5 cylindrical 26,650 LiFePO_4_ battery module is presented in Figure 8 [94]. Figure 8 systematically evaluates the performance boundaries of standalone passive CPCM thermal management systems by separately adjusting three independent variables: discharge rate, external air convection coefficient, and cell radial thermal conductivity. All groups of parametric results converge to a unified core conclusion of diminishing marginal returns for single-parameter optimization.

As presented in Figure 8b, an obvious latent heat plateau can be observed at discharge rates from 1 C up to 3 C. Once the discharge rate exceeds the threshold of 3 C (the region to the right of the gray dashed line), the temperature curve rises sharply. This indicates that passive PCM systems have a rated operating ceiling of 3 C; continuous heat accumulation occurs beyond this limit due to insufficient solidification and latent heat regeneration during operation. Figure 8 reveals a counterintuitive rule: raising the external convective heat transfer coefficient from 5 to 40 W/(m^2^·K) only reduces the maximum temperature by approximately 2 °C, while the inter-cell temperature difference expands by nearly 4 °C. This demonstrates that intensifying external forced air cooling brings negligible cooling benefits and simultaneously undermines the core advantage of PCMs in homogenizing internal module temperature. Figure 8d further identifies saturation behavior at the material level. When the cell radial thermal conductivity exceeds 1.5 W/(m·K), further thermal conductivity enhancement yields marginal peak temperature reduction. Even a tenfold increase in radial thermal conductivity lowers the maximum battery temperature by less than 1 °C. Collectively, the three sets of curves in Figure 8 exhibit highly consistent trends: pure passive PCM systems possess an inherent performance saturation plateau. Optimizing a single performance parameter to its extreme cannot fundamentally eliminate the heat accumulation issue, which necessitates the adoption of hybrid cooling architectures to improve PCM thermal regeneration. These quantitative results provide solid numerical support for the hybrid thermal management schemes introduced in Section 3.2.

Beyond high-temperature heat dissipation, preheating and maintaining battery temperature in low-temperature environments are critical requirements for a battery thermal management system. PCMs, leveraging their latent heat, can also provide thermal buffering and insulation at low temperatures. Coupling PCM with a heating module enables a full-temperature-range thermal management system that addresses both high-temperature cooling and low-temperature preheating. Chen and Zhang et al. [95] integrated a heating plate with PCM as an active thermal management technology for battery packs under low-temperature conditions. The results showed that the PCM significantly delayed the temperature drop and alleviated the temperature gradient within the pack. At an initial temperature of −20 °C, the PCM maintained the battery temperature above 20 °C for 3600 s. Furthermore, the system could preheat the battery from 12.21 °C to 16.55 °C within 20 min at an ambient temperature of −10 °C, and from 13.8 °C to 18.88 °C at 0 °C, demonstrating its suitability for battery preheating and insulation in low-temperature environments.

Overall, hybrid thermal management systems fully leverage the synergistic advantages of different thermal management technologies, overcoming the heat accumulation limitation of single PCM systems while compensating for the poor temperature uniformity and high energy consumption of traditional active cooling systems. This approach represents the most promising technological pathway for the industrial application of PCM-based thermal management. Future research will focus on lightweight and integrated system design, as well as parameter matching and energy efficiency optimization under multi-field coupling [96,97].

From a systematic perspective, pure passive PCM systems are only suitable for moderate operating conditions, while PCM-liquid cooling hybrid schemes deliver the best overall performance for high-rate discharge scenarios. This review identifies that the core of hybrid system optimization is the matching between PCM thermal storage capacity and active cooling power, rather than the unilateral improvement of a single parameter. Nevertheless, current research still lacks adaptive control strategies tailored to dynamic driving cycles, which is a key direction for future system-level development.

## 4. Conclusions and Outlook

This review has systematically assessed the field of phase change material-based battery thermal management from both material and system viewpoints. At the material level, organic phase change materials remain attractive for their high latent heat and chemical compatibility, yet their practical deployment is constrained by low thermal conductivity, unsuitable phase change temperatures, and inherent flammability. Considerable progress has been made in addressing these limitations. The incorporation of carbon-based or metal-based skeletons significantly improves heat transfer, though such enhancements often reduce latent heat capacity. Phase change temperatures can be effectively tuned through chemical synthesis or eutectic blending, enabling adaptation to different operating environments. Flame-retardant modifications, either intrinsic or additive-based, have markedly improved safety by delaying thermal runaway propagation. Moreover, the integration of multiple functions—thermal conductivity, flame retardancy, flexibility, and shape stability—into single composites has emerged as a frontier direction, particularly for vibration-prone automotive applications.

**Figure 9:**
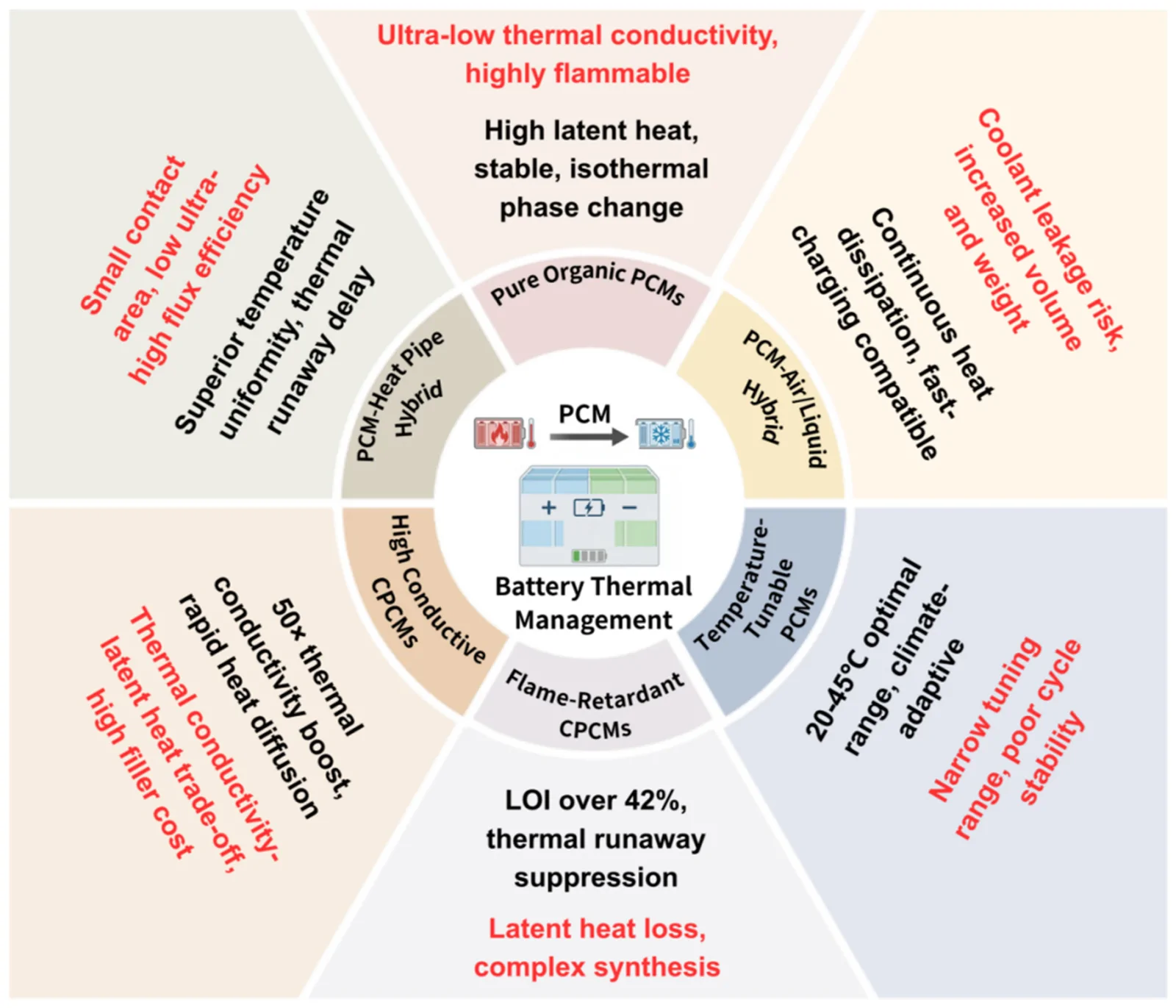
Summary diagram of PCM-based battery thermal management systems, covering material modification strategies, system configurations, and their corresponding advantages and limitations.

To visually organize the full technical landscape discussed in this review, a panoramic summary is provided in Figure 9. It systematically outlines core modification strategies at the material level, typical system configurations at the system level, and the corresponding advantages and inherent bottlenecks of each technical route, serving as a structured reference for researchers and engineers in this field.

At the system level, purely passive phase change material configurations demonstrate clear advantages in peak temperature suppression and temperature uniformity under moderate conditions. However, they suffer from heat accumulation under sustained high-rate operation, which limits their standalone application. Hybrid systems that combine phase change materials with forced air or liquid cooling overcome this bottleneck by actively removing stored heat and enabling regeneration of the phase change material. Among these, phase change material-liquid cooling hybrids offer the most robust performance, maintaining battery temperatures within the optimal range even under aggressive charge–discharge cycles. Additionally, coupling phase change materials with heating modules extends thermal management to low-temperature environments, enabling preheating and insulation.

Looking forward, future development should focus on four interconnected directions. First, multifunctional integrated design should move beyond single-parameter optimization toward composites that simultaneously deliver high thermal conductivity, high latent heat, flame retardancy, flexibility, and energy conversion capabilities. Second, intelligent responsive phase change materials capable of sensing temperature and adapting their thermal properties on demand could substantially improve system efficiency and safety. Third, cost-effective and scalable manufacturing processes are essential to bridge the gap between laboratory innovations and industrial deployment, with increasing attention to bio-derived and salt-hydrate-based materials, along with full-life-cycle environmental impact assessment and closed-loop recycling technologies. Fourth, precise structural engineering—enabled by biomimetic design, nanoconfinement, and interface chemistry—will allow tailorable solutions for specific battery form factors and operating profiles. Addressing these challenges will require close collaboration across materials science, thermal engineering, and battery systems to realize the full potential of phase change materials in next-generation energy storage applications.

## Figures and Tables

**Table 1:** Comparison of different thermal conductivity enhancement strategies.

Enhancement Strategy	Typical Thermal Conductivity Enhancement	Latent Heat Loss	Cost	Advantages	Disadvantages	Applicable Scenarios
Expanded Graphite based	2–10 times	10–15%	Medium	Simple preparation, high efficiency	High density, slightly increased weight	Medium-cost industrial modules, conventional electric vehicles
Graphene/carbon nanotube based	5–50 times	5–10%	High	Ultra-low filler loading, minimal latent heat loss	High preparation cost, easy agglomeration	High-end battery systems, small-scale thermal management
Metal Foam based	3–20 times	15–20%	Medium-High	3D continuous network, rapid heat transfer	High weight, high cost	High-rate discharge scenarios, extreme working conditions
Nano-metal powder based	0.5–2 times	<5%	Medium	Simple blending, easy scale-up	Easy to settle, poor cycle stability	Low-cost low-rate applications

## Data Availability

No new data were created or analyzed in this study. Data sharing is not applicable to this article as it is a review paper that summarizes previously published studies.
